# Possible rheumatoid arthritis subtypes in terms of rheumatoid factor, depression, diagnostic delay and emotional expression: an exploratory case-control study

**DOI:** 10.1186/ar4204

**Published:** 2013-03-21

**Authors:** Taavi Tillmann, Rajeev Krishnadas, Jonathan Cavanagh, KV Petrides

**Affiliations:** 1 University College London, London Psychometric Laboratory, 26 Bedford Way, London WC1H 0AP, UK; 2 University of Glasgow, Institute of Health and Wellbeing, College of Medical, Veterinary and Life Sciences, Southern General Hospital, Glasgow G514TF, UK

## Abstract

**Introduction:**

Dysregulation of the hypothalamic-pituitary-adrenal (HPA) axis has been implicated in the pathology of rheumatoid arthritis (RA), particularly as vulnerable personality types are exposed to chronic stress. Emotions are powerful modulators of stress responses. However, little is known about whether patients with RA process emotions differently to matched controls. In this study we: 1) assessed whether the trait emotional intelligence (trait EI) scores of patients with RA differ from healthy controls at the facet level; 2) explored any subgroups in RA, in terms of trait EI and common risk factors.

**Methods:**

A total of 637 patients with RA were compared to 496 controls on the trait EI Questionnaire (TEIQue). RA subgroups were explored in terms of trait EI, rheumatoid factor status (RF+/-), depression and time from onset of symptoms until diagnosis (diagnostic delay).

**Results:**

The RA group rated themselves lower on Adaptability, Stress-management, Emotion management, Self-esteem, Sociability, Assertiveness, Impulsiveness and Well-being, and higher on Empathy and Relationships than healthy controls. The RF- subtype reported more time with depression (25.2 vs. 11.3 months), a longer diagnostic delay (3.0 vs. 1.7 years), and greater emotional expression (5.15 vs. 4.72), than the RF+ subtype. These differences were significant at the *P *<0.05 level, but not following strict Bonferroni corrections and should therefore be treated as indicative only. RF- patients with a longer diagnostic delay reported depression lasting three times longer (42.7 months), when compared to three other subtypes (11.0 to 12.7 months).

**Conclusions:**

RA patients and controls differ in their emotion-related personality traits, as operationalized by trait EI. These differences may make people with RA more susceptible to chronic stress and HPA-axis dysregulation. RA may be a highly heterogeneous illness where at least two subtypes may be characterized by personality, psychiatric and immunological differences. RF- status, as well as diagnostic delay and emotional expression, may predict future risk of depression. Research on the causes of RA could benefit from a systems science approach.

## Introduction

The causes of rheumatoid arthritis (RA) remain largely unknown. Widespread agreement exists for only three risk factors (female gender, family history of RA and smoking tobacco [1]). These three cannot sufficiently describe an individual's risk of RA, suggesting that other risk factors remain to be discovered [2]. One candidate risk factor for the development of RA is psychological stress [3]. For example, stressful life-events have preceded the onset of RA in as many as 86% of cases [4], and higher stress at the onset of disease predicts worse disease prognosis [5-7]. It has been suggested that people with RA may be hypersensitive to certain stressors and/or generate a bigger stress response [8].

Certain personality traits can amplify the stress response. For example, people with the type A personality have increased levels of cortisol, epinephrine, C-reactive protein (CRP), fibrinogen, Hb_A1C_, high-density lipoprotein/total cholesterol (HDL/TC), systolic blood pressure and body mass index (BMI) [9]. People who report more hostility also show increased levels of epinephrine, norepinephrine, glucose, HDL/TC, systolic blood pressure and waist-to-hip ratios [10]. Low novelty seeking and high harm avoidance have both been linked to increased cortisol secretion in response to the dexamethasone test [11]. Neuroticism and introversion have been associated with a blunted hypothalamic-pituitary-adrenal (HPA) axis response [12]. This all suggests that personality traits may be linked to immune dysregulation, which may be important for autoimmune diseases, such as RA. While we are not aware of studies linking stress biomarkers to the personality of patients with RA, patients with RA have nonetheless been found to be more perfectionistic and neurotic [13] than matched controls. Theory would predict that patients with RA may be more sensitive to negative stimuli and hence more likely to trigger a stress response at lower thresholds [14]. Emotions are powerful modulators of stress-responses, but modern personality inventories often neglect aspects of personality that describe how one feels and processes emotions. There could be value in explicitly exploring the emotion-related personality traits of patients with RA. To our knowledge, no such study has yet been done. Understanding these mechanisms may enable us to better predict the course of RA, and potentially develop specific cognitive behavioural therapies [15-18].

We hypothesized that patients with RA may process emotions differently to controls and decided to investigate this using the personality trait emotional intelligence (trait EI). Trait EI is defined as a constellation of emotional self-perceptions concerning one's abilities to recognize, process and utilize emotion-laden information [19], details of which are seen in the tables. An alternative label for trait EI is "trait emotional self-efficacy". Trait EI should not be confused with seemingly related constructs that try to objectively gauge how "Emotionally Intelligent" one is, using IQ-like tests. Such tests have proved less useful, since it can be difficult to agree on the correct response to emotional questions. However, measuring EI as a *trait *is more straightforward, as assessment is facilitated by self-report questionnaires, where there are no right or wrong answers.

We have recently reported the broader results of a related study where we compared overall trait EI scores of healthy controls to various people with inflammatory conditions [20]. Results showed that patients with RA have only slightly lower scores in overall trait EI (4.90 ± 0.71) when compared to controls (4.97 ± 0.61), a difference that was non-significant in that sample. Significant differences were also found in two of the four trait EI factors, with RA patients rating themselves lower on Well-being and Sociability than controls. The two-fold aim of the present paper is to look more closely at the RA versus healthy group comparison in terms of the 15 specific facets of trait EI, as well as to explore any potential subtypes of RA.

### Subtypes of RA

We explored the possibility of identifying potential subtypes of RA, defined in terms of their trait EI scores. Patients with RA have shown significant inter-individual variation in genotype, gene expression, cytokine patterns, rheumatoid factor (RF) levels, histopathology, microanatomy [21], clinical manifestations and response to treatment [22]. For example, RF is present in only 70 to 80% of patients with RA, and people can be RF+ without developing RA [23]. This literature suggests that RA may be caused by multiple pathways, each of which is expressed in a slightly different manner in each individual [24].

This perspective dictates that all studies on risk factors should look not only for differences between cases and non-cases, but also explore any potential differences in risk factors between subgroups of cases. Researchers in the 1960s to 1980s applied this perspective to the analysis of the rheumatoid personality, which they suggested is composed of two subtypes linked to RF status [25,26]. However, this idea has not been thoroughly investigated in recent literature, so we decided to follow it up by exploring any possible personality differences in trait EI between the RF+ and RF- subgroups.

Most of the recent studies of personality in RA have assumed that there are no pathological subtypes of RA, and that there is only a single disease entity. As a result, their samples would have comprised various combinations of RF+ to RF- patient ratios. This may be responsible for some of the previous discrepancies in results, particularly with regards to anger expression, impulsivity and defiance [27]. We hypothesized that contradictory findings could be resolved at the subgroup level and that when comparing RF+ and RF- subgroups, we may find differences in the trait EI facets of emotion expression, impulsiveness and assertiveness.

### RA and depression

The prevalence of depression in patients with RA is 13 to 42%, which is many times higher than in the general population [28]. Two parallel mechanisms have been suggested to explain this. First, disability from RA prevents patients from functioning the way they used to, thus generating feelings of loss and depression [29]. Second, the proinflammatory cytokine milieu that accompanies acute flares in RA may have a direct neural effect in promoting sickness behaviour and corresponding depressive symptoms. While studies of depression in RA have been inconclusive about the role of such a pathway [30,31], studies in stand-alone depression have found a significant inflammatory component [32], allowing anti-inflammatories to be used as effective adjuncts in the treatment of resistant depression [33,34]. It has been postulated that good treatment of depression in RA may also lead to synergistic improvements in the inflammatory component of RA [35]. Stress-management training has enhanced their cognitive-behavioural variables of self-efficacy, helplessness and coping style, which in turn have led to reduced pain and depression in patients with RA [36,37]. Given the proximity of emotional processing to the themes of stress-management, inflammation and depression [38], we complemented our study with a brief exploration of possible personality differences in trait EI with respect to depression.

## Materials and methods

A cross-sectional study was conducted to compare: a) the trait EI profiles of RA patients against controls at the level of the 15 trait EI facets, and b) the full trait EI profiles between the RF+ and RF- subgroups. The research was conducted in accordance with the Declaration of Helsinki, and ethical approval was granted by the UCL Research Ethics Committee. Patient societies were approached in countries of comparable lifestyle (UK, Ireland, USA, Canada, Australia, New Zealand). Societies in the UK and Canada chose to participate, and emailed approximately 3,000 invitations to their members to complete our online questionnaire. Informed consent was obtained from all participants on our website. A total of 744 questionnaires were received, of which 637 were fully completed. A total of 496 healthy control subjects were randomly drawn from the normative database of the trait EI instrument used in the study (see Table 1 for a description of the samples). We used the Trait Emotional Intelligence Questionnaire (TEIQue) version 1.5 (Manufacturer: K. V. Petrides, London Psychometric Laboratory, London, UK) [39], the psychometric validity of which has been confirmed in many independent studies [40]. Participants respond on a 7-point Likert scale, ranging from 'completely disagree' to 'completely agree'. A total of 153 items are psychometrically combined to derive 15 facet scores, which give rise to four broad factors (Emotionality, Sociability, Self-control and Well-being) and global trait EI.

**Table 1:** Table of demographics for the rheumatoid arthritis and healthy control groups

	Rheumatoid arthritis	Healthy control
***Mean age (standard deviation)***	49.3 (11.2)	41.5 (10.0)
***Gender***		
male	44 (9.4%)	234 (46.8%)
female	424 (90.6%)	266 (53.2%)
***Education***		
basic	107 (23.3%)	62 (12.9%)
technical	121 (26.3%)	85 (17.7%)
university	232 (50.4%)	334 (69.4%)
***Annual Income ***		
<£ 25,000	204 (51.8%)	166 (39.2%)
25,000 to 50,000	144 (36.5%)	153 (36.1%)
>50,000	46 (11.7%)	105 (24.8%)
***Ethnic origin***		
white (U.K.)	301 (64.6%)	310 (66.2%)
white (other)	151 (32.4%)	81 (17.3%)
other	14 (3.0%)	77 (16.5%)
***Marital status***		
single	159 (34.0%)	86 (18.8%)
married	305 (65.2%)	269 (58.7%)
living together	42 (9.0%)	53 (11.6%)

For subgroup analysis, we also asked participants to report brief details of known risk factors: rheumatoid factor status, current smoking status, alcohol intake, age at onset of symptoms and age at diagnosis. Diagnostic delay was calculated by subtracting age at onset of symptoms from age at diagnosis. For ANOVA analysis, diagnostic delay was divided into two categorical variables: patients who took longer than 12 months to be diagnosed from the onset of symptoms (delayed diagnosis), and patients who took less than 12 months to be diagnosed from the onset of symptoms (quick diagnosis). As a rapid estimate of the extent of co-morbid depression, participants were also asked to estimate the total number of months they had been treated for depression throughout their lifetime.

Statistical analysis was conducted with SPSS Statistics 18.0 (Manufacturer: IBM, Armonk, New York, USA). A series of *t*-tests were used to compare the continuous variables (trait EI profiles; age of onset; age of diagnosis; diagnostic delay; depression; alcohol consumption) across categorical groups (patients vs. healthy controls; RF+ patients vs. RF- patients). The Bonferroni adjustment was used to control for Type I error. A series of bivariate Pearson's correlations were used to test for associations between continuous variables. Two-way ANOVAs were used to test for interactive effects between two categorical groups (patients vs. healthy controls; males vs. females; RF+ patients vs. RF- patients; quick diagnosis vs. delayed diagnosis), on continuous dependent variables that reached significance in previous one-way testing (namely the four trait EI factors and the total number of months spent on treatment for depression).

## Results

### Trait emotional intelligence in rheumatoid arthritis vs. controls

Significant differences were found in 8 of the 15 underlying personality facets, with the RA group rating themselves higher on relationships and empathy, and lower on stress management, emotion management, adaptability, assertiveness, impulsiveness and self-esteem (*P *<0.0025 following Bonferroni adjustment, Table 2). There was no relationship between trait EI scores and the number of years the patient had been ill, neither in the RA group as a whole, nor in just those RA patients whose symptoms started within the last five years.

**Table 2:** Trait Emotional Intelligence profile comparison between rheumatoid arthritis and healthy control groups (means; *t*-tests)

*Factor*/Facet	Rheumatoid arthritis (*n *= 637) Mean (SD)	Healthy controls (*n *= 496) Mean (SD)	*t *- value	Effect size (Cohen's *d*)
*Well-being *	5.10 (1.06)	5.27 (0.82)	3.03***	0.18
Self esteem	4.76 (1.04)	4.97 (0.83)	3.79***	0.22
Happiness	5.35 (1.31)	5.55 (1.01)	2.83**	0.17
Optimism	5.18 (1.18)	5.29 (0.98)	1.60	0.10

*Self-control *	4.65 (0.86)	4.66 (0.80)	0.14	0.01
Emotion regulation	4.53 (0.97)	4.56 (0.86)	0.60	0.03
Stress management	4.47 (1.11)	4.69 (0.99)	3.51***	0.21
Low impulsiveness	4.97 (0.97)	4.73 (0.97)	4.05***	0.25

*Emotionality *	5.21 (0.81)	5.08 (0.74)	2.66**	0.17
Emotion perception	4.92 (0.95)	4.89 (0.83)	0.50	0.03
Emotion expression	4.83 (1.36)	4.82 (1.21)	0.10	0.01
Relationships	5.71 (0.82)	5.46 (0.80)	5.26***	0.31
Empathy	5.36 (0.83)	5.15 (0.79)	4.21***	0.26

*Sociability *	4.70 (0.82)	4.95 (0.75)	5.39***	0.32
Social awareness	4.90 (0.99)	5.04 (0.88)	2.37*	0.15
Emotion management	4.59 (0.82)	4.84 (0.85)	4.99***	0.30
Assertiveness	4.60 (1.06)	4.98 (0.87)	6.46***	0.39

Adaptability	4.55 (1.02)	4.74 (0.84)	3.26***	0.20

Self-motivation	5.01 (0.91)	4.89 (0.82)	2.38*	0.14

*Global trait *				
*Emotional intelligence *	4.92 (0.72)	4.97 (0.61)	1.45	0.08

*P (value) <0.05; **P (value) <0.01; ***P (value) <0.0025 following Bonferroni adjustment to control for experiment-wise Type I error.

### Gender interactions

**Figure 1:**
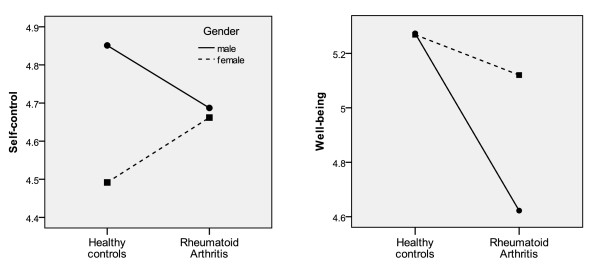
**Interactive effects between gender and group (rheumatoid arthritis vs control) on two emotional intelligence factors**.

We tested for interactive effects between gender (male vs. female) and group (patients vs. healthy controls) on four trait EI factors (Figure 1).

Well-being: Significant main effects were found for gender (F(1,960) = 8.20), *P <*0.01), group (F(1,960) = 21.5), *P <*0.01), as well as their interaction (F(1,960) = 8.53; *P *<0.01). While healthy males and females had comparable scores in Well-being, RA males had significantly lower Well-being scores than RA females.

Self-Control: Significant main effects were found for gender (F(1,960) = 6.67, *P *<0.01), and the interaction (F(1,960) = 5.11, *P *<0.05), but not for group (F(1,960) = 0.003, *P *= 0.96). On Self-control, RA males scored lower than healthy males, while RA females scored higher than healthy females. There were no main or interactive effects with gender on the other two trait EI factors, Emotionality and Sociability.

### Subtypes within the RA group

We asked 286 patients with RA whether they knew their rheumatoid factor (RF) status, of whom 222 patients answered positively. A total of 157 patients (70.7%) had been tested positive for rheumatoid factor (RF+), and 65 (29.3%) had been tested negative for rheumatoid factor (RF-). A total of 292 patients answered concerning whether they had received treatment for depression, of whom 139 (47.6%) had received treatment at some point in their lives, and 153 (52.4%) had never received treatment for depression.

We explored whether the RF+ and RF- subgroups show any differences in terms of their trait EI profiles or simple disease characteristics (Table 3). Of the 20 trait EI scores, the two groups scored very similarly on 13 scores with a non-significant score difference of less than 0.15 points on the Likert scale. On seven scores, small differences (0.15 to 0.3 points) were seen between the RF+ and RF- groups, but these were non-significant (effect size Cohen's *d *= 0.13 to 0.23). By content, these borderline differences suggested that RF- patients may score slightly lower on stress management, optimism, happiness, well-being, impulsivity and slightly higher on assertiveness, when compared to RF+ patients. On the emotion expression score, the difference was more pronounced (0.43 points, *d *= 0.33), with RF- patients scoring higher on emotion expression than RF+ patients. This was statistically significant at conventional levels (*P *<0.05), but not after an experiment-wise Bonferroni correction was applied (*P *>0.0025).

**Table 3:** Comparison of rheumatoid factor + (RF+) and rheumatoid factor- (RF-) subgroups in terms of trait emotional intelligence scores and other characteristics

	RF+ (*n *= 157) mean (SD)	RF- (*n *= 65) mean (SD)	*t *- value	Effect size (Cohen's *d*)
Personality facet *Emotion expression*	4.72 (1.29)	5.15 (1.34)	2.21*	0.33
Personality facet *Optimism*	5.23 (1.19)	4.94 (1.31)	1.60	0.23
Personality facet *Happiness*	5.34 (1.28)	5.04 (1.45)	1.53	0.22
Personality factor *Well-being*	5.11 (1.05)	4.88 (1.17)	1.44	0.21
Personality factor *Emotionality*	5.15 (0.73)	5.30 (0.82)	1.36	0.19
Personality facet *Assertiveness*	4.49 (0.98)	4.69 (1.05)	1.35	0.20
Personality facet *Low impulsiveness*	4.81 (1.01)	5.00 (0.92)	1.33	0.20
Personality facet *Stress management*	4.49 (1.00)	4.35 (1.14)	0.90	0.13
Personality facet *Empathy*	5.31 (0.74)	5.41 (0.87)	0.86	0.12
Personality facet *Social awareness*	4.90 (1.01)	4.79 (1.08)	0.70	0.11
Personality facet *Self-esteem*	4.76 (0.99)	4.66 (1.03)	0.69	0.10
Personality facet *Emotion regulation*	4.47 (0.88)	4.38 (0.98)	0.69	0.10
Personality facet *Emotion perception*	4.90 (0.89)	4.97 (1.01)	0.46	0.07
Personality factor *Sociability*	4.66 (0.85)	4.70 (0.87)	0.35	0.05
Personality facet *Emotion management*	4.58 (0.87)	4.62 (0.88)	0.31	0.05
Personality facet *Relationships*	5.67 (0.78)	5.68 (0.81)	0.11	0.01
Personality facet *Self-motivation*	4.93 (0.94)	4.94 (0.90)	0.10	0.01
Personality factor *Self-control*	4.59 (0.81)	4.58 (0.86)	0.10	0.01
Personality facet *Adaptability*	4.48 (0.98)	4.48 (1.10)	0.03	0.00
Global trait *Emotional intelligence*	4.87 (0.68)	4.87 (0.75)	0.02	0.00

Diagnostic delay (years from onset to diagnosis)	1.71 (3.33)	3.00 (5.23)	2.18*	0.30
Depression (months on treatment)	11.3 (33.0)	25.2 (72.7)	2.06*	0.26
Social class (8 = professional)	5.85 (2.11)	6.28 (1.73)	1.56	0.22
Age	49.1 (10.8)	46.8 (11.1)	1.43	0.21
Age of onset of symptoms	39.1 (12.7)	36.7 (14.6)	1.22	0.18
Annual Income (11 = ≥ £ 50 000)	5.30 (3.00)	5.64 (3.25)	0.70	0.11
Current smoker (1 = yes; 2 = no)	1.87 (0.34)	1.85 (0.36)	0.39	0.06
Exercise (hours/week)	1.75 (2.70)	1.88 (2.89)	0.32	0.05
Gender (2 = female)	1.93 (0.26)	1.94 (0.24)	0.23	0.04
Education (3 = university)	2.34 (0.77)	2.36 (0.78)	0.17	0.03
Alcohol (units/week)	7.52 (10.9)	7.33 (11.7)	0.09	0.02

*P (value) <0.05.

Comparison of other disease characteristics showed that the RF+ and RF- groups were virtually identical in terms of their demographics (education, income) and lifestyle factors (alcohol, smoking, exercise). The RF- group was 2.3 years younger and developed symptoms 2.4 years later than the RF+ group, and had a slight leaning toward higher socioeconomic status. However, these differences were not statistically significant. The RF- groups had spent twice as much time being treated for depression (*d *= 0.26), and waiting nearly twice as long for their diagnosis from disease onset (*d *= 0.30). This was statistically significant at conventional levels (*P *<0.05), but not after an experiment-wise Bonferroni correction was applied (*P *>0.0025).

Two additional correlations were found to be statistically significant, even after a Bonferroni adjustment (*P *<0.0025). Thus, "wait-time from symptom onset until diagnosis" was positively correlated with the number of months spent on treatment for depression (*r *= 0.285), and negatively correlated with the age of onset of symptoms (*r *= 0.186).

**Figure 2:**
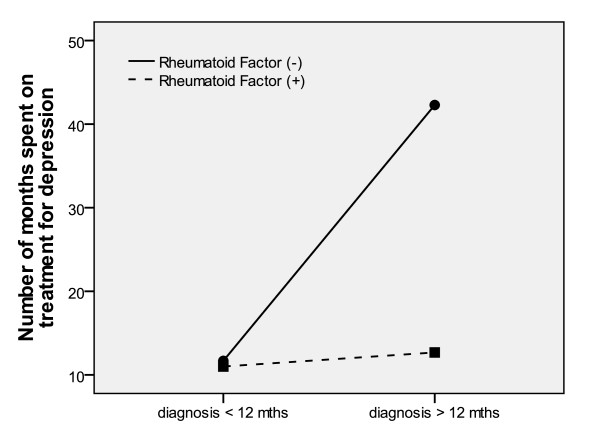
**Mean number of months treated for depression, across rheumatoid factor status and diagnostic delay**.

Subsequently, a two-way ANOVA was conducted with RF status and diagnostic delay as the independent variables, and the number of months spent on treatment for depression as the dependent variable (Figure 2). Significant main effects were found for both RF status (F(1,239) = 4.54, *P *<0.05) and diagnostic delay (F(1,239) = 5.72, *P *<0.05), meaning that the RF- subgroup reported longer treatment for depression than the RF+ subgroup. Those whose diagnosis took longer than 12 months reported more treatment for depression than those whose diagnosis took less than 12 months. We also observed an interactive effect (F(1,239) = 4.87, *P *<0.05), meaning that within the RF+ subgroup, having a long diagnostic delay did not increase the number of months spent on treatment for depression. For the RF- subgroup, however, having a longer diagnostic delay increased the number of months spent on treatment for depression from 12 to 43 months.

## Discussion

We found significant differences in 8 of the 15 trait Emotional Intelligence facets, when comparing RA patients to healthy controls. RA patients scored lower on assertiveness, self-esteem and impulsiveness. This agrees with earlier descriptions of RA patients as shy, self-sacrificing and compliant, suggesting that they place greater priority on the needs of others as opposed to their own needs [41,42]. Such emotion-related personality traits are not fully represented in common personality inventories, such as the Big Five of Openness, Conscientiousness, Extraversion, Agreeableness and Neuroticism. As a result of not looking for this cluster of affective traits, some studies have concluded that no personality differences exist between RA and healthy control groups [43]. Our findings, however, lend support to the conclusions of other studies that have specifically investigated such personality traits.

RA patients reported greater empathy and better relationships, but lower emotion management and stress management than healthy controls. These facets have not been previously investigated psychometrically in rheumatoid populations, but similar themes have been described, including lack of 'emotional maturity', and 'struggling to contain hostile thoughts' [44]. Since low emotional control has been associated with greater levels of pain [45] and worse rehabilitation outcomes [46], this line of enquiry may have the potential to yield clinical benefits in the future.

We also found RA patients to be less adaptable than healthy controls. This trait too has received little attention in the RA personality literature, but corresponds broadly with psychological rigidity [47]. Altogether, these five emotion-related traits have not always been included in personality inventories, which could explain why these differences have not been previously detected. We encourage their inclusion in future studies investigating the personality of rheumatoid patients.

An interesting question is whether these emotion-related personality differences arose as a consequence of having to adapt to life with a debilitating condition or whether they had been present before the onset of disease. Unfortunately, we are not aware of any longitudinal study of personality in rheumatoid arthritis that could help shed light on this issue. Personality is generally stable over time, particularly in the 40 to 60 age group of our participants [48] although it can change under some situations, such as major life events [49,50] or pregnancy [51]. Therefore, the possibility remains that some of these personality differences may have arisen as a consequence of RA. Nonetheless, we feel that our data offer little support for this perspective. For example, if personality changed in response to the illness, we would expect some personality scores to correlate with time spent being ill, particularly during the first few years after onset. However, we found no such correlation either in the RA group as a whole, or in those RA patients whose onset started within the past five years. Although by no means definitive, this suggests that the personality differences we report may have been present before the onset of the disease. Future cohort studies would be best placed to answer this question.

Seven out of eight significant personality differences are consistent with the hypothesis that RA patients are less good at controlling the build-up of chronic stress, compared to healthy controls (relationships was the one facet where the RA group scored higher). Looking at those facets with the biggest effect sizes, the RA group scored lower on assertiveness (*d *= 0.39) and emotion management (*d *= 0.30). It is plausible that less assertive people may develop more internal stress, the ineffective discharge of which via maladaptive emotion management strategies may cause such stress to persist or intensify. Chronic stress may lead to over-activation and dysregulation of the HPA axis, as well as dysregulation of the autonomic nervous system [4]. This, in turn, can lead to inflammatory dysregulation, which may precipitate and perpetuate rheumatoid arthritis in certain individuals [52-54].

### Gender differences

We found that RA males have much lower scores in well-being and self-control than control males. A similar significant difference was found between RA females and control females in well-being, but this difference was significantly smaller in magnitude (Figure 1). To our knowledge, this is the first study to describe personality differences in RA with respect to gender. If these differences arose as a response to illness, it suggests that men are particularly vulnerable to loss of well-being and self-control. Men may benefit more from targeted cognitive behavioural therapy to increase their self-confidence and positive affect, as well as skills of emotional control, stress management and general self-reflection [55]. If these personality differences were present before disease onset, this suggests that men and women develop RA along slightly different pathways, wherein the role of personality manifests itself in a gender-specific manner. Future studies investigating the determinants of RA should explicitly look for gender-personality interactions.

### Significance of RA subtype associations

Many RF+ versus RF- differences reached levels of conventional statistical significance, but these were lost after the application of stringent Bonferroni corrections to control for experiment-wise error. One could take the strict view and dismiss these findings as "not significant". However, we decided to give these borderline associations the benefit of the doubt, and comment on them in our discussion. This is partly because explorative studies should report results that were close to reaching significance, with the intention to encourage further confirmatory studies. Second, our sample size in these comparisons was very small (*n *= 65 and 165 for RF- and RF+ groups), but the mean differences were relatively large, suggesting that larger studies may well confirm these associations as statistically significant. Moreover, some of the associations we uncovered could have been hypothesized by prior theory, after which *P *<0.05 would have denoted statistical significance. For example, literature suggests that RF- people have a greater prevalence of psychological maladjustment than RF+ people, making it plausible for RF- people to spend more time with depression. In any case, while we have elaborated fully on associations where 0.0025 <*P *<0.05, we stress that our data are only indicative, and require replication on larger studies.

### RA subtypes by depression

The lifetime prevalence of depression in our RA group was 47.6% (that is, 47.6% of patients had received some treatment for depression during their lifetime). We are not aware of previous literature describing the lifetime prevalence of depression in RA. However, the lifetime prevalence of depression is usually twice the current prevalence at any given moment [56]. Using this heuristic, we would expect the lifetime prevalence of depression in an RA population to be between 30 to 60%. Our finding of 47.6% falls within this range, suggesting that our sample was representative of the general RA population, in terms of tendency towards depression.

RF- patients reported more time spent with depression than RF+ patients. We are not aware of a similar finding in previous literature [57]. RF- status has, however, been linked to the covariates of depression, such as psychiatric disorders, psychoticism, anxiety and neuroticism [26,58,59]. Furthermore, RF- status has been associated with lower self-acceptance, greater psychological rigidity, obsessive-compulsiveness, somatization complaints, emotional detachment, reduced emotional functionality, social alienation and paranoid ideation [25,60,61]. Our novel finding that RF- patients tend to spend more time being treated for depression is, therefore, consistent with the existing literature profile of higher psychopathy in RF- patients. This suggests that RF, a readily available biomarker, may be a clinically useful risk factor for predicting subsequent depression in patients with RA. This informs the on-going debate about the utility of RF testing in clinical practice [62].

We describe a further subgroup interaction that renders some individuals particularly vulnerable to depression: RF- patients for whom it took longer than 12 months to be diagnosed since the onset of their symptoms, spent a significantly longer period on treatment for depression (mean = 43.2 months), compared to the three other subgroups (RF+, long diagnosis = 12.7 months; RF+, short diagnosis = 11.0 months; RF-, short diagnosis = 11.7 months). This novel finding suggests that the "RF-, delayed diagnosis" subtype may be especially vulnerable to developing recurrent depression, or simply resistant to current methods of treating depression. Again, this subtype is clinically easy to describe, so future studies could explore whether they would benefit from earlier treatment for depression, or a different treatment approach, in order to prevent its recurrence.

### RA subtypes by emotional expression and diagnostic delay

RF- patients in our study reported greater levels of emotional expression than RF+ patients. This matches previous descriptions of RF- patients displaying hostility and aggression more readily than RF+ patients [63]. Emotional expression is also related to emotional disclosure, which has been associated with lower disease activity and better health outcomes in RA [64,65]. In healthy volunteers, emotional disclosure has been linked to short-term reductions in T-helper cells, T-killer cells, total lymphocyte counts [66], greater brain congruence between left and right hemispheres, reduced autonomic stress as measured by skin conductance, as well as fewer somatic complaints [67]. This body of work led us to the hypothesis that the release of psychological stress via emotional expression may protect the immune system (RF- patients). Conversely, RF+ patients who tend to suppress or 'bottle up' psychological stress may be causing some form of neuro-immunological damage. This damage would add to other pathological processes that eventually manifest as rheumatoid factor antibodies in the common, more severe subtype of RA that is easier to diagnose.

If emotional expression gives immunological benefits to the RF- subtype, then how can we explain its association to psychological harm? This could be explained if patients associate frequent emotional expression to notions of failure or unattractiveness. Using psychodynamic theory, we can speculate that people with RA may begin with a common belief that others are more important than themselves [68]. This creates a desire to suppress any self-centred thoughts and feelings [41]. Because such feelings naturally rise in everyday life, we can separate the two RA subtypes depending on how they respond to such situations.

The RF+ subtype could be more successful in meeting the demands of their super-ego, by suppressing their emotions effectively. This reconciliation between the desired self and the actual self can lead to psychological well-being and stability. However, there can be a physical flipside to the coin, whereby the suppression of ego-centric thoughts may have negative consequences on immune dysregulation (either directly or indirectly through mediators like the uptake of smoking). These processes ultimately culminate with the production of rheumatoid factor, more severe rheumatoid arthritis and a quicker diagnosis. Further to the associations described above, RF+ has also been associated with smoking [69], lower socioeconomic status [70], the DRB1*0401 gene [71], melatonin receptor type 1 [72], reduced autonomic cardiovascular reflexes [73], soluble granzymes [74], and reduced soluble receptors for advanced glycation end products [75], all of which leads to increased inflammation, more pentosidine in the synovium [76] and a poorer response to anti-TNFα therapy [77]. It remains to be seen precisely how our findings may relate to these features of the RF+ subtype.

In contrast, the RF- subtype could be less successful at controlling their self-centred emotional expressions. This would create a conflict between the desired self and the actual self, and generate feelings of guilt and remorse that contribute to depression. On the flipside, perhaps it is this subtype's ability to discharge selfish wants and the ensuing guilt, which gives their immune system protection against HPA axis dysregulation and subsequent development of rheumatoid factor [59]. The RF- subtype develops RA along different pathways that are more associated with the DRB1*0301 [78], DRB1*08, DRB1*11, SLC11A1 [79] and *HLA-B27 *[80] genes, the use of the oral contraceptive pill [81], and increased IL-1ss and IL-12p40 cytokines leading to a monocyte-based inflammatory cascade [82]. The RF- subtype is not associated to socioeconomic status. It is important to remember that this discussion is speculative. We provide it only to spur other researchers to consider the wider associations that may be underlying each of the various factors, so that the scientific community may start to move toward a complex but integrated map of the aetiology of RA subtypes.

RF- patients reported a longer delay from the onset of symptoms until a diagnosis was made. This is another novel finding for which we suggest three possible explanations. First, RF- status is associated with milder forms of RA and fewer clinical signs on presentation [83]. This makes the subtype more difficult for clinicians to diagnose, creating the diagnostic delay. Second, RF- patients score higher on measures of neuroticism [59], which renders them more sensitive to physical complaints and more likely to seek medical treatment, even if clinical signs have not yet fully developed. Third, RF- patients are more likely to report somatization symptoms [26], knowledge of which may bias the general practitioner to misdiagnose the presentation as a somatization disorder.

### Limitations

Our study has three main limitations. First, while we used a comprehensive, validated instrument to assess trait emotional intelligence, we were unable to employ validated instruments to assess depression or rheumatoid factor status. For these two variables, we used a rapid method of self-assessment by single question only. This was dictated by the nature of our research, which was explorative/formative, rather than definitive/summative. Second, while the differences between RA versus controls reached strict significance, those between RF+ and RF- subtypes lost their significance after Bonferroni adjustment. Thus, our findings should be considered as indicative and would need to be replicated on larger samples with a full battery of validated instruments. Third, our sample was not stratified, but limited to members of patient societies. Furthermore, our response rate was only 25%, so the sample might be weighted toward those who spend more time on the internet, those who are more comfortable with submitting data over the internet, or those who are particularly interested in personality and emotionality. Future research should make an effort to recruit from a broader base of eligible patients.

## Conclusions

While previous work may have suggested that RA patients do not differ to healthy controls in their global trait EI scores, this paper suggests that they do score differently on eight specific trait EI personality facets. Each of these may, in theory, leave them vulnerable to chronic stressors and potential HPA axis dysregulation. While the exact mechanisms are still unclear, emotion-related personality traits appear to play a role as modulators or co-factors in the complex cascade of events that trigger the pathogenesis of RA. Accordingly, future longitudinal studies investigating the causes of RA would benefit from incorporating trait EI personality factors alongside primary variables of interest.

Our exploratory data suggest that RA patients may divide into two subtypes. The majority group is RF+, characterized by low depression, low emotional expression and a short wait time from onset until diagnosis. The minority group is RF-, with high depression, high emotional expression and a longer wait time from onset until diagnosis. These findings are in keeping with the perspective that RA is a highly heterogeneous disease.

The etiological mechanisms of RA may be better understood if future studies explicitly make the assumption of heterogeneity, and take a systems science approach to study design in order to assess as many variables as possible and to include as many subjects as possible. This would enable researchers to zoom in on the pathological pathways behind ever-smaller subtypes, allowing us to ultimately personalize the management and prevention of each subtype accordingly. For subgroup analysis, researchers should assess gender, depression, rheumatoid factor, delay time from onset until diagnosis and a variety of personality traits (emotional expression, psychoticism and neuroticism may be particularly useful when studying RF+ and RF- subgroups). These variables could be studied alongside physical variables, such as genes, measures of stress sensitivity and reactivity (for example, HPA axis response, skin conductance, EEG congruence or heart rate variability), and cytokine profiles or cytokine responsivity patterns, in order to develop a systematic and comprehensive understanding of the role of stress in causing and perpetuating RA.

## Abbreviations

ANOVA: Analysis of variance; BMI: Body Mass Index; CRP: C-reactive protein; EEG: Electroencephalography; HBA1C: Glycated haemoglobin; HDL: High-density lipoprotein; HPA axis: Hypothalamic-pituitary-adrenal axis; IL: Interleukin; IQ: Intelligence quotient; MANCOVA: Multivariate analysis of covariance; RA: Rheumatoid arthritis; RF: Rheumatoid factor; SD: Standard Deviation; TEIQue: trait EI Questionnaire; TNFα: Tumour Necrosis Factor alpha; Trait EI: trait emotional intelligence; TC: Total cholesterol; UCL: University College London

## Competing interests

The authors declare that they have no competing interests.

## Authors' contributions

TT conceived of the study, collected the RA data, performed the subtype analysis and drafted the manuscript. RK contributed to statistical analysis, the psychological interpretation of data and revised the manuscript. JC participated in the study design, contributed the biological interpretation of data and revised the manuscript. KVP designed the study protocol, coordinated its execution, collected the control data, performed the core analyses and revised the manuscript. All authors read and approved the final manuscript.
